# Adolescents’ use of an online Q&A service to enhance their oral health literacy: a mixed-methods text mining and thematic analysis

**DOI:** 10.1186/s12903-026-07848-z

**Published:** 2026-02-04

**Authors:** Eva Lassemo, Kari Sand, Randi Krog Eftedal, Cecilie Robertsen, Marikken Høiseth

**Affiliations:** 1 Department of Health Research, SINTEF Digital, Trondheim, Norway; 2 Department of Diversity & Inclusion, NTNU Social Research, Trondheim, Norway; 3 Center for Oral Health Services and Research, Mid-Norway (TkMidt), Trondheim, Norway; 4 The Public Dental Services Trøndelag County Authority, Steinkjer, Norway; 5 Department of Design, Faculty of Architecture and Design, Norwegian University of Science and Technology (NTNU), Trondheim, Norway

**Keywords:** Oral health literacy, Adolescent, Health information, Information seeking, Decision-making, Online Q&A service

## Abstract

**Background:**

Health literacy is fundamental to healthy behaviour, defined as seeking, processing, and evaluating health information to make informed decisions. Access to health information has historically been linked to socioeconomic status (SES), with disparities in health persisting due to unequal access to new health technologies. Digital technologies offer opportunities for spreading health information but may also increase inequalities through a digital divide. The Norwegian government established the website ung.no in 2003 to provide health information to adolescents. This study aims to investigate what oral health information adolescents seek on the ung.no Q&A service and how they use it to build oral health literacy.

**Methods:**

The study analysed 1632 questions posted on the ung.no Q&A service and answered by dentists between January 1, 2020, and May 12, 2023. Data included self-reported gender and age of the individuals posting the questions. Descriptive analyses, text mining, manual coding and qualitative thematic analysis were used to assess the four elements of health literacy: finding, processing, evaluating and utilizing health information.

**Results:**

The analysis revealed that most questions were posted by girls (65.8%), with the majority coming from the 13–15 age group (55%). Common themes included orthodontic treatment, caries, oral hygiene, finances and the impact of diet and substances on oral health. Adolescents used the Q&A service to seek information, confirm information from other sources, and evaluate options for oral health behaviours and treatments. Many questions reflected concerns about the appearance of teeth, fear of dental procedures and the confidentiality of dental visits. Negative emotions such as stress, fear and shame were prevalent, impacting the adolescents’ ability to process information and make informed decisions.

**Conclusion:**

The ung.no Q&A service is a valuable tool for adolescents to increase their oral health literacy. The study highlights the importance of providing clear, accessible information and addressing the emotional factors that influence health behaviours. Dental professionals should focus on building trust, offering empathic communication, and providing proactive guidance to support adolescents in making informed decisions about their oral health.

## Background

Health literacy is fundamental to healthy behaviour. Health literacy could be defined as seeking, processing and evaluating health information and adequate use of the acquired information in health-informed decision-making [1].

Historically, access to health information has been linked to education, income, power, and social capital, i.e., socioeconomic status (SES) [2–4]. The social gradient in health and mortality has been argued to persist based on individuals experiencing lower SES being less able to take advantage of new health technology. Because education tends to enhance the willingness and ability to exploit technological advances, technological progress may serve to further increase disparities in health [5]. The introduction of fluoridated toothpaste has been used as an example of health technology more likely to be adopted by people of higher education, income and SES [6].

Digital technologies and the internet facilitate opportunities for global spread of public health information as well as medical treatment not requiring physical contact. However, digital health comes with the paradox that it potentially may contribute to further increasing already existing inequalities through a digital divide [7]. Globally, great differences exist both between availability and use of the internet [8].

Young people’s use of digital technology to find, understand and use health information is characterized by their preference for the internet over traditional sources, such as personal consultations, and their prioritization of online content over other references [9, 10]. As digital natives, they have grown up with technology and are accustomed to integrating it into their daily lives [9, 10]. Despite this, they frequently seek support from parents and healthcare professionals to understand the information they find online [9].

Adolescents use the internet and social media platforms to seek health information, with Google being the most popular search engine [11, 12]. However, they rarely assess the quality of the content they encounter and often lack the skills to critically evaluate its quality and reliability [13]. It has been reported that information adolescents find online can lead to behavioural changes [14]. Previous research on young people’s use of digital services does not include a question-and-answer (Q&A) service similar to ung.no which forms the basis for analysis in our study [11, 12].

Regarding oral health literacy specifically, Stephens, Ryan [12] investigated how adolescent orthodontic patients sought information and found that while most patients (84%) located information about future treatment by talking to either a general dentist or to an orthodontist, only a small fraction (8%) searched the internet al.though 64% reported using the internet daily. Over recent years we have seen a surge in the internet as information source and while 99% use the internet daily, 79% of 16–24-year-olds in Norway used the internet to search for health information within the previous three months [15].

To provide universal and directed information to all adolescents, the Norwegian government through the Norwegian Directorate for Children, Youth and Family Affairs (Bufdir), established the website ung.no in 2003 geared towards 13–20-year-olds. One of the features in ung.no is an anonymous Q&A service where users can either search a database of over 500,000 previous questions with answers or ask their own questions which are in turn answered by professionals. The service is widely used; in 2021 for the first time, more than 100,000 questions were asked from children and adolescents in one year, which were answered by the approximately 200 professionals associated with ung.no. The large database containing all previous questions and answers is a rich source of information for young people when they themselves search for answers and can also give clear indications of what adolescents are interested in and who is interested in what. Descriptive analysis of over 100,000 questions written to ung.no from 2016 to 2018 showed that most youth asked questions about body and health [16]. While adolescents often turn to fast-paced platforms such as social media, search engines, or peer forums for health information, the Q&A service on ung.no offers a distinct alternative. It is publicly funded, professionally moderated, and anonymous, and does not rely on peer-to-peer interaction or influencer content. Its growing popularity suggests that adolescents value access to trustworthy, confidential, and expert-based health information tailored to their concerns [17]. This positions ung.no as a unique hybrid between formal health communication and youth-oriented digital engagement.

A survey among young conscripts of the Norwegian Armed Forces completing their first-time military service suggested that the respondents had lower oral health literacy than the general population [18]. Despite this, we still know relatively little about adolescents’ oral health literacy, specifically, what kind of oral health information they look for, how they seek it out, and how they evaluate this information when making decisions about their own oral health. Thus, the aim of the present study is to investigate what information, within oral health, adolescents are seeking on the ung.no Q&A service, and, how adolescents are using the ung.no Q&A service to build oral health competency.

## Methods and materials

### Setting

In Norway, the Norwegian Public Dental Service (PDS) provides free dental care, except most orthodontic treatment, for children (0–18 years of age) and at discounted rates for older adolescents aged 19–20 and young adults aged 21–24 (as of 2023) [19]. Nearly all, 98.2% of children aged 1–18 utilize the free PDS [20]. Based on the cost brackets according to age, it is not uncommon to plan potentially expensive treatments, such as extraction of wisdom teeth, before crossing into a costlier bracket.

### Data

The data consisted of all questions posted on ung.no and answered by a dentist in the period January 1st, 2020, through May 12th, 2023, totalling 1632 questions. The dataset consists of naturally occurring, user-generated questions submitted anonymously by adolescents to the ung.no Q&A service. Unlike self-reported reflections collected through interviews or surveys, these questions represent adolescents’ actual communicative practices in a real-life setting. This provides a unique opportunity to explore how adolescents engage with oral health information, express uncertainty, seek reassurance, and navigate sensitive topics in their own words.

Data were obtained from Bufdir. Available data on each question is self-reported gender and age of the individual posting the question, a timestamp of when the question was posted, in addition to the question itself. Due to the anonymous nature of the Q&A service, nothing can be known about the representativeness of the adolescents using it. The service is targeting adolescents in the ages 13–20 years old. The dataset consists of texts that are available on the internet, but that were not originally produced for the purpose of research. The data are anonymous and contain no person identifiable data or sensitive information. The nature of the data is such that tracing it back to the adolescent producing it is impossible; hence the authors of the questions have not been asked to give consent. Under Norwegian law, anonymized data are not personal data and fall outside the Personal Data Act (2018) and GDPR; therefore, consent and ethical approval were not required. Ethical considerations, governing the use of data and texts from ung.no in research were adhered to as described by Lassemo, Sand [21].

### Analyses

#### Descriptive analyses

Through structured and unstructured text-mining analyses we have generated data on the data. These are data that statistically describe the material. Examples of this is number and share of questions distributed on posters’ age and gender, number and share of groups of questions distributed on posters’ age and gender, development of extent of submitted questions over time, and co-occurrence of themes in questions.

#### Literacy analysis

The data were analysed by applying text mining, manual coding, and qualitative thematic analysis to assess the four elements of health literacy: finding, processing, evaluating, and utilizing health information to make informed choices. To determine how adolescents use the Q&A service to find information, we used text-mining and manual content coding to identify what young people seek information about, while qualitative thematic analysis was used to interpret how they process, evaluate and utilize the information.

#### Text-mining

Unstructured text can contain unimaginable amounts of information not represented in structured or coded elements. Using natural language processing or text-mining, patterns and knowledge can be extracted from text documents. A central part of text-mining is to connect the information from large amounts of text data to discover and form new concepts, connections between words or sentences (semantic relations), as well as new facts and new hypotheses for further investigations [22]. Text-mining is a variation of a method known as data mining, which seeks to find interesting patterns in large databases. Methodologically, text-mining is based on a mixture of methods from various fields, including language processing, statistical methods, machine learning, reasoning and knowledge management [23].

To get an overall overview of what young people seek advice and guidance about on ung.no within the topic of oral health, we carried out a text-mining analysis of all submitted questions. To carry out an analysis of this size, we used the text analysis program WordStat [24] in combination with the statistical computer program STATA [25] and Excel.

Data were prepared for analyses by applying stop-words, such as articles, pronouns and prepositions, and stemming and lemmatization, a process of reducing words to their root form. Due to the sometimes informal or speech-like language used in questions, error correction was an important part of data cleaning. A Norwegian dictionary was used for the data preparation and analyses.

Frequencies by which words are used in the text corpus are used to provide an overview of the texts’ content. Categorization in text-mining is done by grouping words having similar meanings. This is a manual process undertaken by the researchers, thus there is no one correct way of categorizing words and hence text.

#### Manual thematic coding

The method we chose for content coding can be described as open thematic coding where we started with an empty code list and added new categories of codes as they appeared. Two researchers together developed the code list and coded the 1632 questions. Themes/codes were not mutually exclusive; each question was given between one and three of the in total 15 codes. This implies that the total number of questions thematically coded exceeds the number of questions posted on the Q&A-service.

The content coding was based on the questions’ content. A significant share of questions contained multiple themes; these were coded to the theme(s) the researchers deemed to be most prominent. Any discrepancies were discussed until consensus was reached.

#### Qualitative thematic analysis

A selection of 756 questions from seven of the themes (53% of the questions in the seven themes) identified in coding were analysed by means of qualitative thematic analysis. Such an analysis involves a close reading of the selected questions to find patterns in theme, communicative functions and forms of expression across the data set. The seven themes were selected based on the highest frequency of questions and to ensure a broad representation of topics and tonal variations in the queries, while the number of questions per theme was determined based on the principle of saturation.

The analysis was carried out in four stages: (1) an initial review of the selected questions to gain an overview of the content, term usage, information needs, communicative functions, and tone of voice; (2) based on the initial overview, establishment of preliminary codes designed to demonstrate how the adolescents use the Q&A service to increase oral health literacy; (3) systematic coding, including adjustment of the codes; and (4) condensation of codes into categories that is in line with the health literacy definition, and categories indicating potential barriers to improving one’s health literacy.

The presentation of the categories includes paraphrases of questions (that is, a form of retelling) or artificial quotations, that is, quotations that have either been changed from the original or combined from several similar questions. Direct reproductions of questions have been avoided for ethical reasons, although most of the questions are available online. The Results section is presented according to the oral health literacy definition and shows how adolescents use the Q&A service at ung.no to seek, process and evaluate oral health information in their decision-making.

## Results

**Fig. 1:**
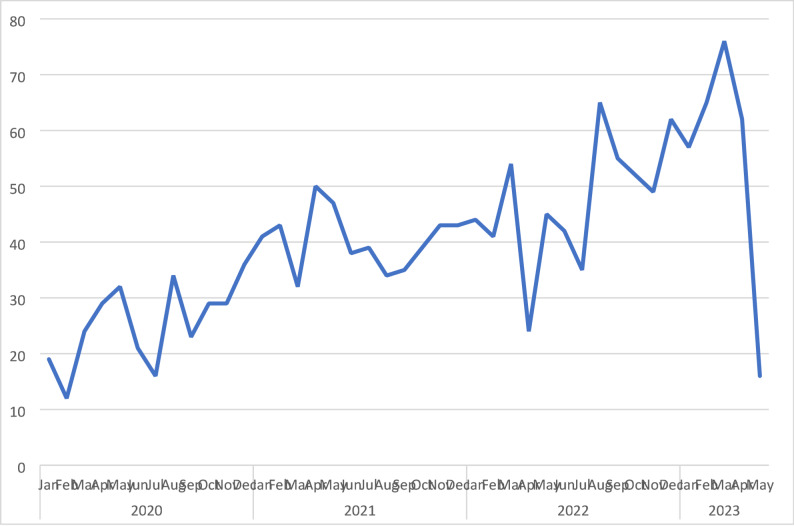
Number of questions posted by month

In total 1634 questions regarding oral health were posted on the ung.no Q&A service in the period January 1st, 2020, through May 12th, 2023. Of these, one question was empty, and one contained no question and thus not possible to code. Analyses were performed on 1632 questions. As can be seen in Fig. 1, there was a clear trend showing an increasing number of questions month by month (note; we only had data from the first 12 days of May 2023).

With little variation over the three and a half years period, questions from girls constituted about two thirds of the data, questions from boys constituted about one third of the data, and with a small fraction posted by adolescents selecting “other” gender (Table 1).

**Table 1:** Absolute and relative number of questions posted, by gender and year

Year	Boys		Girls		Other		Total
	*N*	%	*N*	%	*N*	%	Total
2020	96	31,6%	200	65,8%	8	2,6%	304
2021	151	31,3%	312	64,6%	20	4,1%	483
2022	175	30,8%	378	66,4%	16	2,8%	569
2023	76	27,5%	190	68,8%	10	3,6%	276
Total	498	31,6%	1081	65,8%	54	2,6%	1632

The younger adolescents ask the most questions, with 55% of questions stemming from the 13–15-year-old age group. Questions from the groups 16-17- and 18–20-year-olds constituted 24% and 22% respectively (Table 2).

**Table 2:** Absolute and relative number of questions posted, by age group and year

Year	13–15		16–17		18–20		Sum
	*N*	%	*N*	%	*N*	%	
2020	172	57%	67	22%	65	21%	304
2021	238	49%	121	25%	124	26%	483
2022	335	59%	129	23%	105	18%	569
2023	150	54%	67	24%	59	21%	276
Total	895	55%	384	24%	353	22%	1632

Using the text mining tool WordStat, we found that the most commonly used phrases by 13-20-year-olds posting questions on the Q&A service were related to cavities, toothache, appearances and a desire for orthodontic treatment, and whether the dentist can detect forbidden/prohibited behaviour, such as underage substance or alcohol use and sexual activity.

Through text mining we identified questions containing specified words (see appendix) related to pain, conditions, ailments or diseases, and to diet and food items. Particularly questions containing words related to diet and food items were coded into many different themes, dependent on the context they were used in, such as orthodontic treatment, “What could it be…”, appearances and practicalities (see Table 3).

**Table 3:** Thematic coding of questions posted, by gender and age group

Gender:	Boy			Girl			Other			Total
Themes: Age spans:	13–15	16–17	18–20	13–15	16–17	18–20	13–15	16–17	18–20	
1. Orthodontic treatment	45	25	19	183	51	31	5	2	1	362
2. Caries	10	5	4	40	18	3	0	0	1	81
3. Finances	16	19	39	44	53	40	2	3	2	218
4. “What could it be…”	42	19	22	87	40	46	2	0	1	259
5. Hygiene	23	13	9	35	26	13	1	0	1	121
6. Appearances	30	22	8	114	48	23	1	1	1	248
7. “Will the dentist see/tell…”	55	12	1	60	25	4	10	1	0	168
8. Afraid	9	7	11	51	20	27	4	3	1	133
9. Practicalities	42	17	26	82	28	40	7	3	4	249
10. Substances	65	15	14	51	27	19	10	1	1	203
11. Becoming a dentist	1	1	0	0	0	1	0	0	0	3
12. What can I do	16	19	5	82	28	24	3	2	1	180
13. The dental appointment	7	6	8	29	16	21	1	1	2	91
14. Pain	10	6	7	42	12	11	1	1	1	91
15. Dental erosion	6	1	4	4	9	7	0	0	0	31
Total	377	187	177	904	401	310	47	18	17	2438

### Finding information: using the Q&A service to seek oral health knowledge

Based on open thematic coding, a total of 15 codes or themes were identified. Table 3 provides an overview of the types of oral health information adolescents in our sample sought when using the ung.no Q&A service.

Not only were girls much more likely to use the Q&A-service more than boys, but the focus of questions also varied by gender. The most common theme in questions from boys was substances including alcohol and tobacco (theme 10). Regardless of gender, the majority of these questions are concerned with whether the dentist will be able to detect that the adolescent has been snuffing/smoking and whether this will be reported to parents (theme 7). It is apparent that adolescents in general are aware that snuff use may cause harm to gums. A common concern is how much snuff use it takes to be harmful.

The thematically most common questions posted by girls, and thus overall, are concerned with orthodontic treatment (theme 1). Within the broader theme orthodontic treatment, there are groups of questions focusing on subthemes such as a desire to get braces; a fear of having to get braces; whether specific food items may be eaten while wearing braces; the cost of braces; loose or missing brackets; lost/worn/not used aligners/retainers.

The possibility of having caries and cavities (theme 2) was a concern to many adolescents, and there appears to be some misconceptions. It is not uncommon to believe that tooth discoloration or dark spots on teeth are in fact cavities. Tooth enamel erosion, or dental erosion (theme 15), is another worry among many adolescents. Many include in their questions an awareness of the detrimental effect low pH drinks like sodas or energy drinks may have on enamel. Yet they express low willingness to change their drinking habits. Some are seeking guidance on how much/how often acidic drinks it takes to be harmful. In relative terms, more older adolescents are concerned with dental erosion, what causes it and what, if anything, can be done to remedy it. Many show a good understanding of what may cause damage to their teeth but still ask for solutions to avoid damage while continuing the damaging behaviour.

Many questions on oral hygiene (theme 5) are practical in nature, e.g., should one brush teeth before or after breakfast; what the best toothpaste is; are home-remedies for tooth whitening dangerous; should one floss before or after brushing. There are also adolescents who for various reasons, e.g., due to poor mental health, have not brushed their teeth regularly or at all for prolonged periods of time. These adolescents typically want to know whether new and improved habits can save their oral health, or if it is too late.

Appearances (theme 6), also of teeth, are of great importance for many adolescents. Among the adolescents’ appearance-related concerns in the questions are: discoloration of teeth and gums, crooked teeth, need for orthodontic treatment, homemade remedies for tooth whitening, altering the size and shape of teeth. Quite a few are expressing feelings of low self-confidence related to oral health issues. Among the most common concerns were discoloration, spots and whitening. Some want to fix what they perceive to be a problem themselves, either by remedies like baking soda, bleach, or they ask advice on what they can safely use. Another common concern was a perceived deviance in the appearance of their teeth, as in a gap between the front teeth (diastema), overbite, underbite, and skewness in bite or tooth/teeth. In general, adolescents want to know whether their perceived problem may be fixed, and whether it comes at a cost (theme 3).

Adolescents display concern regarding whether what they are experiencing is potentially dangerous or something that they should worry about (theme 4 and 8). The things they worry about range from a slight discoloration of a tooth or gum, to thinking they may have an oral cancer or HIV. Other things adolescents are afraid of are cavities, dental erosion, needles, needing braces, and tooth extraction. Not knowing what will happen or what a given treatment entails causes fear for many. Some of the adolescents have experienced being humiliated by their dentist and ask for guidance on how to overcome their fear in order to complete future needed treatment.

Questions regarding practicalities are also common (theme 9). Among the subthemes emerging were: a tooth has become loose, should it; how can I know the difference between a baby tooth and an adult tooth; wisdom teeth; will X behaviour affect my teeth? Another subtheme is questions or concerns the poser either forgot to or did not dare ask during a resent dental appointment. Many of these are concerned with orthodontic treatment.

While the questions on practicalities, and most of the rest, tend to be focused on gathering facts or learning how something works, or who can aid in solving a problem, there was a group of questions focused on what the adolescents themselves can do to better their situations. Some have had braces, now they are removed, and the way their teeth look is back to how they were. Common to most of these questions is: “X has happened, what can I do to fix it?”.

Questions centered around finances or costs of dental care (theme 3) change character with the increasing age of those asking. In the youngest group, 13–15-year-olds, a typical cost-related question is regarding the cost of braces, or if tooth whitening is a free service for those under 18. There are many teenagers wondering whether they can get cosmetic treatments, like teeth whitening, covered by the PDS. Some appear well-informed and have questions about future costs of care given a specified condition or ailment. Among the 16–17-year-olds questions about at what age they will need to start paying for what services emerge. Among the oldest group, this is the most common theme of questions. The 18–20-year-olds want to know how much they have to pay for what; if some services still are free or discounted; if they have any rights to free treatment now if the need is based on deferral from the dentist or they previously have received sub-par treatment.

The proportion of questions concerning the dental appointment (theme 13) increases by age. A number of adolescents pose questions on the theme of how they can know who their dentist is, how/if they can switch to a different dentist, how they can make an appointment and whether they need to wait to be called for an appointment. As the Covid pandemic was within the period our data stems from, there are a number of questions related to Covid; whether the clinic will be open, how Covid affects availability of treatment, as well as hygiene measures at dentist offices. Experiences with rude, rough or unwelcoming dentists and staff are presented in questions asking for guidance on how to handle this. Negative experiences with dental appointments are either based on a fear of dentists or have caused a fear of dentists (cf. theme 8).

In terms of questions regarding pain (theme 14) there are essentially two categories; those who are experiencing pain and those who are afraid of contracting pain. Among the first category are those who for some time have been experiencing some discomfort in the oral cavity or have recently gotten braces or retainers. In the second category, are those who soon have a dental appointment and are concerned about pain from the treatment they will undergo.

Another theme displaying a clear age gradient is “will the dentist be able to tell… and will (s)he let my parents know?” (theme 7). While there are hardly any questions coded in this theme posted by the oldest group, it is quite common among the youngest. Posters from the youngest group typically have smoked, used snuff, or done other illegal actions, and are worried their parents will get to know either directly by being present at the consultation or indirectly by the dentist informing them later.

In the “What can I do” category (theme 12), we find a mix of questions. Some are looking for do-it-yourself solutions to dental concerns, some are looking for guidance as to whether they should seek professional help, at this point or later, while some express desperation of greater or lesser extent, rhetorically asking what to do. Problems raised in this theme include loose teeth (is it a baby tooth or a permanent tooth? ) and orthodontic treatment not providing desired results. Another concern seen among the older group is the cost of treatment. Several are posting questions asking advice on what to do, given that they are perceiving a dental problem and afraid they will not be able to cover the costs.

### Understanding information: using the Q&A service to process oral health content

When using the Q&A service to process information, adolescents often seek confirmation of information they have heard from others or discovered independently. Frequently, the information they require clarification on originates from their dentist. The need for confirmation or further explanation of information, whether self-sourced or received from others, is evident across various topics, however, it is particularly pronounced in the context of “cavities” where many users seek clarification on the fundamental nature of cavities, such as their appearance and formation.

Quite a lot of questions arise from the adolescents’ personal observations and experiences. In their questions, they often describe specific dental conditions, such as spots, streaks, or dots (frequently black), discoloration, or pain and sensitivity, and inquire about their potential association with cavity development. The following question is an example of the need to understand what a cavity really is:

*“Two of my bottom front teeth are really stained and have like a hard coating on the bottom part of them. There’s also another tooth that kind of stings when I bite on a certain spot. I’m worried it might be cavities or something else that needs to be drilled” (Girl*,* 13)*.

Further, regarding cavities, the adolescents’ questions indicate a significant confusion surrounding the concept of “incipient cavities”, and thus a need for clarification of this phenomenon, as the following example illustrates:

*“Two years ago*,* my dentist said I almost had a cavity*,* but last September*,* they said I didn’t. I was so sure I had one since they said the same thing before! I thought that was weird*,* haha. Is it normal to almost have a cavity and then go back and they say it’s gone?” (Girl*,* 15)*.

### Evaluating information: using the Q&A service to assess relevance and reliability

When adolescents use the Q&A service to evaluate information, they seek additional knowledge to make informed choices about their oral health, i.e., they use the Q&A service as a decision support tool. This is particularly evident in questions coded “What could it be/is it dangerous?“. On the one hand, the questions in this theme suggest a good level of oral health literacy among the adolescents, as they demonstrate awareness of potential oral health problems, understand the need for reliable information to identify the cause, and can differentiate between self-treatable issues and those requiring a dentist’s expertise (potentially caused by their own actions). On the other hand, the adolescents use the Q&A service to get an evaluation of the different plausible alternative explanations they are already aware of, e.g., the most likely diagnosis (is this plaque or something else? ), the underlying cause (braces or snuff? ), and the appropriate course of action (dentist visit or self-monitoring? ).

The adolescents’ questions also show the need for evaluation of available options regarding own behaviour or healthcare use. They write that they have some available options, which they have found or been given, and they use the Q&A service to seek assistance in evaluating these options. They want to understand the outcomes of each choice, determine what is the best option for them, or explore additional alternatives because they are either unwilling or unable to choose from the options they already know. The following question illustrates a need for help evaluating two options to make an informed choice: Here, the alternatives are (1) replacing one tooth at the dentist or (2) faking an accident to lose the tooth on purpose:

*“I’m really not happy with my top teeth. Now that I’m 17*,* it’s super embarrassing to have braces*,* especially since everyone else is getting them off. There’s one tooth that really sticks out and looks bad. Can I get a dentist to replace it*,* or should I fake an accident and get it pulled out? I know some people who have damaged their teeth and gotten a new one.” (Boy*,* 17)*.

When using the Q&A service to help make decisions about their oral health, the adolescents often seem to seek knowledge to support their own arguments. This would allow them to engage in discussions with parents or dentists and actively participate in decision-making. By doing so, they position themselves as informed users/patients who want to make their own choices, rather than simply following the advice of dental professionals. This behaviour is particularly evident in questions related to braces. The adolescents ask about their options, such as whether they can choose to have braces or not, whether they can start with braces on the lower jaw only, whether there are alternatives to braces, and if they can have the braces removed earlier than recommended by the dentist.

In the question below, a girl who has been given the option to have braces, needs assistance to develop a rationale for her decision. She wants to know more about why the dentist recommends braces and what the implications are if she chooses to have them or not.

*“I’ve got crooked teeth and I’m wondering if I have to get braces. My dentist said I could choose*,* but they’d probably get braces if it was them*,* just because they’re dentists. Is it going to mess up my jaw later if I don’t fix my crooked teeth?” (Girl*,* 16)*.

### Applying information: using the Q&A service to make informed oral health decisions

The choices discussed in these questions primarily fall into two categories: (1) decisions about personal oral health behaviours (such as what to do, what should be done, or what is best) and (2) decisions about seeking dental care.

Many questions related to contacting dental services, such as “Will the dentist find out and tell my parents?”, reveal a need for more information about what to expect at the dental office and about patients’ rights, including dental professionals’ duty of confidentiality. In some cases, this knowledge seems crucial for adolescents to even consider visiting the dentist.

A specific type of question concerns whether the dentist can detect signs of substance use (such as snuff, vaping, smoking, alcohol, or drugs), sexual activity, or eating disorders and whether the dentist can disclose this information to the adolescent’s parents. Adolescents express a critical need to know this information to attend their scheduled dental appointments. They fear the consequences of their parents finding out about their behaviour and convey their stress and anxiety through phrases like ‘please reply quickly,’ ‘crisis,’ ‘please help,’ ‘I’m scared,’ ‘really scared,’ and ‘terribly scared.‘. The following question demonstrates the urgent need for information and its impact on a teenager’s decision to attend a dental appointment:

“*Hey, I’m 15 and I’ve been using snuff quite a bit, but I don’t think it’s caused any damage besides my gums receding a little. I have an appointment with an orthodontist next week to see if I need braces, and my mom wants to come with me. Will the orthodontist bring it up? If he definitely brings it up, I’ll have to cancel the appointment, which I really don’t want to do. Orthodontists aren’t like regular dentists, so maybe he won’t mention it?*”

### Negative emotions as a mediating factor for increased oral health literacy

On the one hand, the range of questions in this sample from the Q&A service demonstrates that many adolescents possess a high level of health literacy: they are actively seeking information, have multiple information sources, critically evaluate information, and engage in a thorough process to make informed choices. On the other hand, the questions are remarkably characterized by negative emotions which can hinder health literacy and the ability to enhance it and make sound choices. Adolescents describe stress, worry, despair, and fear – emotions that impact one’s ability to find, understand, and evaluate information, as well as make informed decisions about behaviours or seeking dental care.

## Discussion

### Main findings

This study aimed at investigating how adolescents used an online, public and quality-assured Q&A service to increase their oral health literacy, i.e., how they seek, process, evaluate and use information to make informed decisions about oral health. The sample of questions is partly characterized by a good number of young people showing high level of oral health literacy and partly characterized by negative emotions such as stress, fear and shame. The adolescents seem to use the Q&A service to increase their own health literacy as a basis for making good choices about their own oral health behaviour as well as for navigating the oral health care service.

The adolescents’ use of a Q&A service is, in itself, a demonstration of a certain level of health literacy, as it reflects ability to identify and access the service and actively engage with professionals by posing questions. Hence, the service supports development of oral health literacy. Orthodontic treatment and appearance were among the two most common topics young people sought information about. The most common issues they needed help with were clarification about whether something they experienced in their mouths or with their teeth is normal or dangerous; whether the dentist can tell their parents about undesirable behaviour such as tobacco use; and what they themselves can do to save a situation or take better care of their oral health.

Adolescents used the Q&A service to seek information, affirmation and reassurance. They asked for help to evaluate available options about behaviour or healthcare or for clarifications about issues such as patient rights and confidentiality. It appears that they wanted to use these affirmations and assessments to be better prepared for navigating the oral health services and for oral health behaviour change. Moreover, there seems to be a correlation between increasing levels of health literacy and the complexity and length of questions, i.e., that the adolescents demonstrated extensive knowledge about oral health, available alternatives, expected behaviours, and navigating the healthcare system through their detailed and lengthy questions.

A general impression from our sample questions is that adolescents demonstrate a high degree of oral health literacy, including the ability to critically evaluate information. This high level of health literacy among adolescents can be attributed to their access to a wide range of information channels through extensive use of the internet and social media to seek health information [26], high level of digital literacy [27], including ability to evaluate reliability of various online sources [28] or a motivation driven by a desire to take care of their own health and to be attractive [29]. However, our analysis indicated that instead of using the information or clarifications to make good decisions for their oral health, adolescents often look for workarounds that allow them to continue behaviours they know to be harmful. Examples include reducing the risk of dental erosion while continuing to drink large amounts of acidic beverages or chewing gum and eating hard foods while wearing braces. Others seek the threshold up to which unhealthy behaviour is still acceptable, such as how much snuff is harmful, how frequently acidic drinks can be consumed without causing harm, or how little the retainer can be used while still being effective. The fact that some individuals persist in behaviour that incur harm to themselves is in line with previous research. For instance, a recent study by Zeng, Park [30], identified three phenotypes; *sensitive*, those who adapt their behaviour based on understanding consequences; *unaware*, those who did not infer causality from experience but corrected their behaviour based on information; *compulsive*, those who persisted harmful behaviour despite both experience and information. The adolescents posting questions as described in the above would fall in the *compulsive* phenotype. This line of behaviour is also in accordance with findings from a recent study demonstrating the difficulty of implementing change in adolescent dental patients [31].

There are several possible explanations for why people, including adolescents, continue unhealthy behaviours despite knowing the risks. Adolescents (and adults for that matter) do not necessarily translate their knowledge into action, in particular not in the long run [32]. Health behaviour is influenced by multiple factors beyond mere knowledge of risks. Factors such as perceived threats, barriers, self-efficacy, social norms, and perceived control also play a significant role [27]. Adolescents often engage in both healthy and unhealthy behaviours simultaneously, demonstrating more behavioural heterogeneity than anticipated [33, 34]. In emotional or high-risk situations, adolescents are more likely to rely on environmental cues and impulses rather than rational judgment based on health knowledge [35]. Adolescents’ health decisions can be influenced by their low perception of risk [36], that their ability to regulate own behaviour is still developing, limited experience with decision-making and few worries for the future consequences of their actions [35].

While the range of questions demonstrates a high level of health literacy – critical evaluation of information from multiple sources and informed decision-making – negative emotions like stress, shame, worry and fear are frequently reflected in the questions. These emotions can hinder adolescents’ ability to process information effectively, impacting their capacity to make sound choices about dental care. Emotions influence decision-making, and in health contexts, shame can significantly impact health choices, potentially limiting discussions of alternatives [37]. Conversely, negative emotions such as fear and anxiety, can also serve as motivators, prompting people to prioritize their health and well-being [38]. Previous research confirms that appearance often seems to be an underlying motivation for many adolescents. In a qualitative study of Midwestern adolescents, Calderon and Mallory [29] found that the factors most prominently influencing oral health behaviours were the desire to be attractive or “kissable”, while the understanding of “healthy” was good looking and attractive to peers rather than having healthy teeth. Studying motivations and expectations concerning orthodontic corrective treatment, Prado, Previato [39] found attractiveness to be the focus for most adolescents considering or undergoing orthodontic treatment.

Although young people are considered digital natives, they still face barriers to digital participation such as lack of access [26] or low self-efficacy [40]. While ung.no is a public, quality-assured information service that is generally accessible to all young people in Norway, there can still be challenges related to the actual accessibility and use of such services. In a review of global youth perspectives on digital health promotion, Ferretti, Hubbs [41] point out that issues related to costs and socio-cultural barriers hindering access to technology are rarely discussed in research, and state that this does not reflect the realities for many young people, especially in low- and middle-income countries (LMIC), due to lack of infrastructure, expensive apps and data subscriptions, low health and digital literacy, and unmet basic needs. These challenges can however also partially be obstacles in high-income countries. Norwegian studies have identified several barriers to digital participation for children and adolescents, including lack of digital literacy, lack of access to equipment and internet, poor ICT equipment often due to low income, insufficient support and competence from parents, negative social control, and overcrowded living conditions that result in a lack of privacy and lack of digital literacy [26, 42, 43].

The Norwegian platform ung.no represents a model for digital health promotion that could inspire similar initiatives internationally. As a government-operated and quality-assured Q&A service, *ung.no* offers adolescents a safe, anonymous, and judgment-free space to ask sensitive health questions and receive evidence-based answers from qualified professionals. This combination of accessibility, credibility, and anonymity lowers barriers to seeking help and fosters trust in health services. When adapted to local languages and cultural contexts, such platforms could effectively support youth health literacy in other countries. Moreover, analyzing submitted questions provides valuable insights into young people’s concerns, allowing health professionals to design targeted, data-driven interventions. By doing so, digital services like ung.no may contribute to strengthening health literacy, enhancing equitable access to care, and reducing social inequalities in health.

### Clinical implications

Our results may provide valuable insight to inform future youth health promotion strategies and priorities as part of the PDS mandate.

To improve health literacy, providing proactive guidance throughout childhood may be essential. The results from our analysis of how adolescents use an anonymous Q&A service, can be useful for dental professionals providing oral health education in various settings, in dental clinics as well as in schools. Merely the fact that adolescents have many questions underscores the importance of prioritizing preventive and health-promoting efforts. The themes identified as relevant for adolescents can be used to tailor oral health education, for instance by informing about these topics, even if the young person does not directly ask about them.

Further, quite a few of the questions are characterised by shame or fear, which indicate aspects of oral health that may be particularly difficult or sensitive for young people. Dental professionals can use this knowledge to become more aware of these issues when interacting with young people, both individually in the clinic and in more general informational settings such as in schools. Adolescents also express concerns about pain, procedures and dentist interactions. Offering detailed explanations of procedures and pre-visit consultations can help manage concerns and anxiety.

Our study suggests that adolescents use the Q&A service not only for information seeking but also more actively as part of decision-making for instance by seeking support for their arguments to make choices, such as weighing options for orthodontic treatments. Dentists should engage in open discussions that empower patients to make informed decisions.

Adolescents use a variety of information channels – with various purposes and in different situations – to search for health information [9, 11, 12]. To ensure broad reach among children and youth, it is important to regularly reinforce basic information while providing opportunities to ask questions and receive support in evaluating situations and making informed choices. While the dental clinic serves as a central arena, home, school, leisure activities and social media can also function as key everyday spaces that collaborate to strengthen this process.

Some of the questions in our study suggest a need for an anonymous and discrete platform as the Q&A service where it is safe to ask embarrassing, difficult, dangerous, or perceived foolish questions. Oral health professionals can assist adolescents in navigating the various available information channels, enabling them to choose sources that meet their needs for quality-assured information and confidentiality. An anonymous Q&A service is valuable but should be complemented by opportunities for personal follow-up, along with carefully designed interactive resources and tailored information, to promote deeper understanding and support informed decision-making.

As discussed above, and underscored by a recent study, changing an unfortunate habit will often meet resistance [31]. The oral health service should focus on various tools in communication and relationship building. From clinical experience we know that a good relationship between dental personnel and adolescents is a key factor in promoting effective preventive knowledge development. A good relationship ensures that the patient adheres to further treatment measures and takes better care of their oral health. Having the knowledge, competence and the skills to build a good professional relationship makes oral health professionals more confident in their role as health promoters and will help to create a sense of security and trust [44]. There are many reasons why establishing good habits and maintaining good oral health can be difficult. It is important that the oral health service meets patients with empathy, engages with their experiences, and shows curiosity and genuine interest in their feelings and perspectives on the problem area [45, 46].

Oral health professionals need to stay updated on the signs and implications of potentially harmful behaviour such as the use of tobacco, including snuff, vaping and smoking, substance abuse and eating disorders, as adolescents seek guidance on these topics. Dentists and dental hygienists should actively educate patients about the long-term implications of these behaviours and support change through careful design of realistic, incremental steps and communication tools tailored to patients’ needs.

Transparency between dental personnel and patients is critical for effective, safe and personalized oral care, ensuring accurate diagnosis and treatment. Patients who feel embarrassed about their oral health may avoid disclosing details about their habits or health history for fear of being judged or reprimanded. A history of critical or unsupportive comments from healthcare providers can heighten patients’ reluctance to share personal information, fearing a repeat experience. Stigmatized behaviours such as substance abuse or poor diet can be especially difficult to disclose, as patients worry about being labeled or misunderstood. Negative emotions such as stress, shame and fear are common among adolescent patients. Patients who feel ashamed or anxious may find it hard to establish trust with their dentist, making it less likely they will share sensitive information. A distant or overly clinical approach from the dentist or dental hygienist can exacerbate this issue. Empathic communication can help alleviate these emotions, fostering better interaction and treatment adherence [47–49]. To encourage transparency, it is vital to establish trust and foster open, non-judgmental communication, and to create a safe and supportive environment for the adolescents. Open communication fosters trust, encouraging and empowering patients to follow through with recommendations and treatment plans [50]. When patients feel heard and understood, they are more likely to adopt preventive measures and healthier habits [46]. Further, dental personnel should educate without criticism, focusing on solutions and improvements rather than what a patient “should” have done differently in the past. Positive reinforcement such as recognizing patient effort in maintaining oral health and overcoming fears can encourage continued engagement with dental care.

The results indicate that adolescents expressed a need for information about patient rights, health professionals’ confidentiality obligations, legal frameworks relevant to children and youth and professional ethics. Many of the questions concerned whether dental health personnel can detect forbidden behaviour (e.g., underaged vaping or sexual activity) and more importantly whether they may inquire about said behaviour in front of parents present during the consultation, or whether they may report on said behaviour in later dialog with parents. This suggests that many adolescents lack knowledge on patient-provider confidentiality, which ensures that information shared during the dental appointment will not be disclosed to others. While educating youth on confidentiality is not the sole responsibility of health personnel, it is a role they should contribute to more actively. To ensure appropriate educational frameworks and sufficient time for discussion, youth in a Norwegian study suggested that dental health personnel recognize schools as a suitable arena for reaching young people [51].

Our findings may help dental professionals develop greater awareness of how to appropriately receive and respond to information about inappropriate behaviour. To address concerns that sensitive topics may arise when parents are present, the dental health service could consider structuring appointments in two parts: one in which parents are included and one in which the adolescent is seen alone. Such an approach may facilitate more open communication, enabling young people to ask questions and provide candid feedback related to inappropriate behaviour.

In many western countries, there is an increase in self-reported mental health problems among adolescents. A report based on interviews and surveys of adolescents in Oslo found a correlation between mental health problems and body image pressure [52]. The Norwegian Dental Association (NDA) has, together with the Norwegian Medical Association, released a policy document addressing cosmetic treatment in medicine and dentistry, stating that “Cosmetic treatment, without a medical or dental indication, is not compatible with good, professional practice and should not be offered by general practitioners or dentists.” [53]. NDA elaborates this by referring to the NOVA report [52] and stressing that dentists should not contribute to increased body pressure in society. At the same time, the sometimes-blurred transition between necessary health care and cosmetic treatment is acknowledged. In many cases, treatment decisions will be based on discretionary assessments made by the individual dentist, considering ethical principles for each individual case. Adolescents often link oral health and appearance to self-esteem. This imposes a delicate balancing act on the dental personnel. Dentists and dental hygienists should take the adolescents’ concerns seriously, and at the same time advocate for a healthy, “normal” mouth and take care not to contribute to enhancing body image pressure, offering both clinical and psychological support when necessary.

### Strengths and limitations

Qualitative categorization of text is performed by the researchers. Had another team of researchers included other words or categorized differently, the results might have been different. This represents an uncertainty, or limitation, by the method. However, the uncertainty is minimalized by two researchers independently coding all questions into themes, and by allowing each question to be coded into up to three themes. Since the categories are not mutually exclusive, a question may fall into one, two or three categories.

Nothing is known regarding the representativeness of the adolescents posting questions on the Q&A service. The only available information about those posting questions is self-reported gender and age. Considering how the Q&A service functions, there is no way of knowing how many unique individuals are using it. Although it is evident that some questions are replications of previously submitted questions, we chose not to exclude any (but the two as reported in results). The reasoning behind this was that the number of questions explicitly stating that this is a replication (due to e.g., lost code for checking answer) was fairly low. It is plausible that even though questions are similarly worded, asked by a person of same age and gender and within few days of each other, they could be asked by separate individuals. Furthermore, the main purpose of this paper was not to quantify accurately the number of unique questions within certain categories. In an insight analysis of ung.no, analysing near 300,000 questions, a similar distribution of age and gender was found as compared to this project [54]. We thus consider including all questions in analyses was warranted.

The time-period the data stems from includes the Covid-19 pandemic. This has likely had some impact on the types of questions asked. E.g., how to make an appointment, or whether the dental clinic will be open.

In this fast-paced world, some questions may already be dated, whereas more current trends emphasizing the importance of “jawline” and “mewing” potentially could have become themes had questions posted until end of 2024 been included. However, upon randomly sampling some questions on these topics, they all are answered by public health nurses, not dentists.

Another limitation of the study is the Norwegian setting and service, which entail some unique characteristics. However, the content of the main topics is likely to be relevant from an international perspective and can contribute to a better understanding of what young populations seek to know about oral health, such as fear of caries, dental erosion, or other serious health conditions, such as HIV or cancer. In countries without similar services, introducing a comparable service could be a health-promoting strategy to reach and engage young people.

Finally, it is important to emphasize that our data set consists of questions, not adolescents. As previously mentioned, due to how the Q&A service is set up, there is no knowing how many individual adolescents have used the ung.no Q&A service to gain oral health competence in the time period. Furthermore, the data included are questions, not answers. Neither do we know whether the answers were ever read by those posing them, nor whether the adolescents benefitted from the answers they received.

Despite these limitations, a key strength of the study is the use of naturally occurring, user-generated questions, as described in the Methods section. By analysing adolescents’ own formulations in a real-life digital setting, we gain insight into how they actually engage with oral health information. This allows us to observe communicative practices as they unfold, offering a more authentic understanding of oral health literacy in practice.

## Conclusions

Our study demonstrated that ung.no plays a crucial role in supporting adolescents’ oral health literacy. Adolescents actively used the platform to seek, validate, and evaluate information related to oral care and treatment options. Common themes and concerns included caries, oral hygiene, finances, the impact of diet and substances on oral health, the appearance of teeth, fear of dental procedures, and the confidentiality of dental visits. Notably, emotional factors such as fear, stress, and shame frequently emerged, affecting adolescents’ ability to make informed decisions. A key clinical implication is the need for dental professionals to recognize and address these emotional factors. Hence, our findings underscore the importance of empathic communication and trust-building in dental care, as well as the need for accessible, youth-friendly health information online.

## Data Availability

The data that support the findings of this study are available from Bufdir but restrictions apply to the availability of these data, which were used under license for the current study, and so are not publicly available. Data are however available from the authors upon reasonable request and with permission of Bufdir.

## Appendix

- Conditions, ailments or diseases of the oral cavity
  - Acid
  - Acid damage
  - Acidic
  - Dental disease
  - Enamel
  - Enamel damage
  - Gingivitis
  - Gum disease
  - Periodontitis
  - Scald
- Diet/food items
  - Apple
  - Beer
  - Bread
  - Breakfast
  - Bris [carbonated water]
  - Caffee
  - Candy
  - Caramel
  - Carbonated
  - Chai
  - Chilly nuts
  - Chips
  - Chocolate
  - Christmas cookies
  - Cider
  - Coconut
  - Cola
  - Crisp bread
  - Diet
  - Dinner
  - Drank
  - Drink
  - Eat
  - Energy bar
  - Energy drink
  - Falafel
  - Food
  - Food plan
  - Fruit
  - Granola
  - Gummy candy
  - Honey
  - Juice
  - Lamb ribs
  - Leftovers
  - Lemon
  - Lemon juice
  - Lemonade
  - Mango
  - Nibble
  - Orange
  - Orange juice
  - Overeating
  - Pancakes
  - Paprika
  - Peanut
  - Pepsi (Max)
  - Pizza
  - Pizza crust
  - Potato chips
  - Raspberry
  - Sea salt
  - Snack
  - Soda/pop
  - Suckers
  - Sugar
  - Tea
  - Water
  - Wheat bun
- Mental health
  - Anxiety
  - Anorexia
  - Bulimia
  - Confidence
  - Depression
  - Psychological
  - Ritalin
  - Self-confident
  - Self-harm
  - Self-image
  - Suicidal thoughts
  - Vomited
- Pain
  - Anaesthesia
  - Hurt
  - Pain
  - Pain killer
  - Painful
  - Sedated
  - Toothache

## Abbreviations

NDA: Norwegian Dental Association
PDS: Public Dental Service
Q&A: Question and Answer
SES: Socio Economic Status
